# Course Control of Underactuated Ship Based on Nonlinear Robust Neural Network Backstepping Method

**DOI:** 10.1155/2016/3013280

**Published:** 2016-05-18

**Authors:** Junjia Yuan, Hao Meng, Qidan Zhu, Jiajia Zhou

**Affiliations:** College of Automation, Harbin Engineering University, Harbin 150001, China

## Abstract

The problem of course control for underactuated surface ship is addressed in this paper. Firstly, neural networks are adopted to determine the parameters of the unknown part of ideal virtual backstepping control, even the weight values of neural network are updated by adaptive technique. Then uniform stability for the convergence of course tracking errors has been proven through Lyapunov stability theory. Finally, simulation experiments are carried out to illustrate the effectiveness of proposed control method.

## 1. Introduction

Tracking control performance for surface vessel along the predefined route has been an essential control problem for marine autopilot system design, and it has received considerable attractions from control community. In 1922, proportional-integral-derivative (PID) autopilot for ship steering was presented by Nicholas Minosky [1]. PID controller greatly improved the performance of autopilots. Until the 1980s almost all makes of autopilots were based on these controllers. One challenge for tracking control of surface vessel based on above method is that the systems are often underactuated by the sway motion due to weight, complexity, and efficiency considerations and exhibit nonholonomic constraints, which meets Brocket's theorem that there is no continuous or even smooth time-invariant state feedback law that can stabilize the system to the origin [2]. Another challenge is that the vessel model itself exhibits severe nonlinear characteristic and model uncertainties induced by the ocean environment [3, 4].

For the ship with nonlinear maneuvering characteristics and without uncertainties, a state feedback linearization control law was designed [5], while feedback linearization with saturation and slew rate limiting actuators was discussed [6]. Later, combined with a genetic algorithm, the backstepping method was employed to develop a nonlinear ship course controller by Witkowska and Smierzchalski [7], where the ship course parameters were automatically tuned to the optimal values with the aid of a genetic algorithm. Even considering the ship steering model with both constant parametric uncertainties and input disturbance with unknown bound, a robust adaptive nonlinear control law was presented based on projection approach and Lyapunov stability theory [8]. Recently many papers have tackled these problems based on Lyapunov theory [9–12]. In [13–15] a global tracking controller for underactuated ship is addressed with nonzero off-diagonal terms, the reference trajectory is generated by using a virtual target guidance algorithm, and the controller designed is facilitated by an introduction of changing the ship outputs, several coordinate transformations, and backstepping method. And the controller design is heavily depending on accurate dynamic model; the robustness against disturbance has not been addressed. A method using backstepping adaptive dynamical sliding mode control is presented for path following control of USV in [16], the control system takes account of the modeling errors and disturbances, and simplified tracking error dynamics are obtained by assuming that the sway velocity is small which can be neglected in the controller design and only for straight line path tracking can be achieved. The LOS based guidance law is also used in the controller design which causes the complexity of computing high-order derivative of virtual control. In [17], a transformation of vessel kinematics to the Serret-Frenet frame is introduced by exploring an extra degree of freedom by controlling explicitly the progression rate of the virtual target along the path and overcomes the major singular problem; approach angle is introduced for controller design via backstepping method. Neural networks are introduced to enhance system stability and transient performance, which can handle the known dynamics and uncertainties of systems well [18–20]. Particularly in [12] a single hidden layer neural network (SHLNN) is adopted to obtain the adaptive signal online, but the choice of the single hidden layer neural network is limited by the number of hidden layer node selections that will affect the online learning speed and accuracy and cannot produce a better estimation effect on the fast changing disturbances.

Therefore, a solution to the course control of underactuated surface vessel is addressed in this paper. In view of the characteristics of the underactuated performance, the backstepping control method is used to deal with above problem. The direct adaptive neural network is adopted to design control law by using the RBF neural network to overcome the problem that the ideal virtual control cannot be used directly in practice. The weights of the neural network are updated by adaptive technique to guarantee the stability of the closed-loop system through Lyapunov stability theory. Simulation results are illustrated to verify the performance of the proposed adaptive neural network controller with good precision.

## 2. Adaptive Robust Neural Network Controller Design

### 2.1. Problem Description

Consider the following nonlinear systems:

(1) x˙i=fix−i+gix−ixi+1+di,1≤i≤n−1,x˙n=fnx−n+gnx˙nu+dn,n≥2,y=x1,

where ${\overset{-}{x}}_{i}=\left[x_{1},x_{2},\ldots ,x_{i}\right]$ is system state, *u* is control input, and *y* is system output. The control objective is to design an adaptive neural network controller and make *y* track *y*_*d*_. *y*_*d*_ meets the smooth bounded reference model as follows:

(2) x˙di=fdixd,1≤i≤myd=xd1,m≥n,

where *x*_*d*_ = [*x*_*d*1_, *x*_*d*2_,…,*x*_*dm*_]^*T*^ ∈ *R* ^*m*^ is state constant, *y*_*d*_ ∈ *R* represents system output, and *f*_*di*_(·),  *i* = 1,2,…, *m*, denote nonlinear function, assuming that the reference model for each state is bounded as *x*_*d*_ ∈ *Ω*_*d*_,  ∀*t* ≥ 0.

**Assumption 1 .**

There is an unknown constant *p*_*i*_^*∗*^ to meet, $\forall ({\overset{-}{x}}_{n},t)\in R^{n}\times R^{+}$,  $\left|d_{i}({\overset{-}{x}}_{n},t)\right|\leq p_{i}^{*}\rho_{i}({\overset{-}{x}}_{i})$, and $\rho_{i}({\overset{-}{x}}_{i})$ is a known positive smooth function.

### 2.2. Direct Adaptive Neural Network Controller Design

In view of the problems and solutions described in the last section, the direct adaptive neural network controller for nonlinear systems with RBF neural network is chosen. Detailed design steps will be described in the following.

**Step 1 .**

Let *z*_1_ = *x*_1_ − *x*_*d*1_, *z*_2_ = *x*_2_ − *α*_1_, and then

(3) $$\begin{array}{c} {\dot{z}}_{1}=f_{1}\left(\;x_{1}\;\right)+g_{1}\left(\;x_{1}\;\right)x_{2}+d_{1}-{\dot{x}}_{d1}. \end{array}$$

Consider the following Lyapunov function:

(4) $$\begin{array}{c} V_{1}=\frac{1}{2g_{1}\left(x_{1}\right)}z_{1}^{2}+\frac{1}{2}{\tilde{W}}_{1}^{T}\Gamma_{1}^{-1}{\tilde{W}}_{1}, \end{array}$$

where ${\tilde{W}}_{1}={\hat{W}}_{1}-W_{1}^{*}$, *W*_1_^*∗*^ represents the ideal weight vector of neural network, ${\hat{W}}_{1}$ represents the estimated value of the neural network weight vector, ${\tilde{W}}_{1}$ represents the estimation error of weight vector, Γ_1_ = Γ_1_^*T*^ > 0 is the adaptive gain matrix, and the derivation of *V*_1_ can be computed as

(5) V˙1z1z˙1g1x1+g˙1x1z122g12x1+W~1TΓ1−1W^˙1=z1g1x1f1x1+g1x1x2+d1−x˙d1+g˙1x1z122g12x1+W~1TΓ1−1W^˙1=z1z2+α1+f1x1−x˙d1g1x1+z1d1g1x1+g˙1x1z122g12x1+W~1TΓ1−1W^˙1.

According to Assumption 1, we can get

(6) V˙1z1z2+α1+f1x1−x˙d1g1x1+z12ρ122g12x1+P1∗22+g˙1x1z122g12x1+W~1TΓ1−1W^˙1=z1z2+α1+f1x1−x˙d1g1x1+z1ρ122g12x1+P1∗22+g˙1x1z122g12x1+W~1TΓ1−1W^˙1.

There is an ideal virtual feedback control law:

(7) $$\begin{array}{c} \alpha_{1}^{*}=-c_{1}z_{1}-\left[\;\frac{f_{1}\left(x_{1}\right)-{\dot{x}}_{d1}}{g_{1}\left(x_{1}\right)}+\frac{z_{1}^{}\rho_{1}^{2}}{2g_{1}^{2}\left(x_{1}\right)}\;\right], \end{array}$$

where *c*_1_ > 0 is designed controller parameter.

Because of the unknown smooth functions *f*_1_(*x*_1_) and *g*_1_(*x*_1_), we cannot actually get the ideal feedback control law *α*_1_^*∗*^; from (7) we can see that the unknown part $\left(f_{1}\left(x_{1}\right)-{\dot{x}}_{d1}\right)/g_{1}\left(x_{1}\right)$ is smooth function of *x*_1_ and ${\dot{x}}_{d1}$, so that

(8) h1Z1≜f1x1−x˙d1g1x1+z1ρ122g12x1,Z1≜x1,x˙d1T⊂R2.

RBF neural network *W*_1_^*T*^ *S*_1_(*Z*_1_) is used to approximate the unknown function *h*_1_(*Z*_1_), and *α*_1_^*∗*^ can be expressed as

(9) $$\begin{array}{c} \alpha_{1}^{*}=-c_{1}z_{1}-W_{1}^{*T}S_{1}\left(\;Z_{1}\;\right)-e_{1}, \end{array}$$

where |*e*_1_| ≤ *e*_1_^*∗*^ is estimated error and meets *e*_1_^*∗*^ > 0.

Because *W*_1_^*∗*^ is unknown, the virtual control law is selected as follows:

(10) $$\begin{array}{c} \alpha_{1}=-c_{1}z_{1}-{\hat{W}}_{1}^{T}S_{1}\left(\;Z_{1}\;\right) \end{array}$$

and then

(11) V˙1≤z1z2−c1z12+g˙1x1z122g12x1+z1e1+P1∗22−W~1TS1z1+W~1TΓ1−1W^˙1.

Adaptive law can be chosen as follows:

(12) $$\begin{array}{c} {\dot{\hat{W}}}_{1}={\dot{\tilde{W}}}_{1}=\Gamma_{1}\left[\;S_{1}\left(\;Z_{1}\;\right)z_{1}-\sigma_{1}{\hat{W}}_{1}\;\right], \end{array}$$

where *σ*_1_ > 0 and then

(13) V˙1≤z1z2−c1z12+g˙1x1z122g12x1+z1e1+P1∗22−σ1W~1TW^1.

Let *c*_1_ = *c*_10_ + *c*_11_, where *c*_10_ > 0 and *c*_11_ > 0, and then the upper equation becomes

(14) V˙1≤z1z2−c10+g˙12g12z12−c11z12+z1e1+P1∗22−σ1W~1TW^1.

According to the complete square formula,

(15) −σ1W~1TW^1−σ1W~1TW~1+W1∗≤−σ1W~12+σ1W~1W1∗≤−σ1W~122+σ1W1∗22,−c11z12+z1e1−c11z12+z1e1≤e124c11≤e1∗24c11.

Because $-(c_{10}+({\dot{g}}_{1}/2g_{1}^{2}))z_{1}^{2}\leq -(c_{10}-(g_{1d}/2g_{1m}^{2}))z_{1}^{2}$, we can make (*c*_10_^*∗*^≜*c*_10_ − (*g*_1*d*_/2*g*_1*m*_^2^)) > 0 by choosing the appropriate *c*_10_ and obtain the following inequality:

(16) V˙1≤z1z2−c10∗z12−σ1W~122+σ1W1∗22+e1∗24c11+P1∗22.

The cross coupling *z*_1_ *z*_2_ in (16) will be eliminated in the next step.

**Step 2 .**

Let *z*_2_ = *x*_2_ − *α*_1_; then

(17) $$\begin{array}{c} {\dot{z}}_{2}=f_{2}\left(\;{\overset{-}{x}}_{2}\;\right)+g_{2}\left(\;{\overset{-}{x}}_{2}\;\right)x_{3}+d_{2}-{\dot{\alpha }}_{1}. \end{array}$$

From (10) we can see that *α*_1_ is a function of *x*_1_, *x*_*d*_, and ${\hat{W}}_{1}$, and  ${\dot{\alpha }}_{1}$ can be written as

(18) α˙1∂α1∂x1x˙1+∂α1∂xdx˙d+∂α1∂W^1W^˙1=∂α1∂x1g1x1x2+f1x1+φ1,

where $\phi_{1}=(\partial \alpha_{1}/\partial x_{d}){\dot{x}}_{d}+(\partial \alpha_{1}/\partial {\hat{W}}_{1})\left[\Gamma_{1}(S_{1}(Z_{1})z_{1}-\sigma_{1}{\hat{W}}_{1})\right]$ can be calculated.

Consider the following Lyapunov function:

(19) $$\begin{array}{c} V_{2}=V_{1}+\frac{1}{2g_{2}\left({\overset{-}{x}}_{2}\right)}z_{2}^{2}+\frac{1}{2}{\tilde{W}}_{2}^{T}\Gamma_{2}^{-1}{\tilde{W}}_{2}, \end{array}$$

where Γ_2_ = Γ_2_^*T*^ > 0 is an adaptive gain matrix.

Then the derivation of *V*_2_ can be calculated as

(20) V˙2V˙1+z2z˙2g2x−2+g˙2x−2z222g22x−2+W~2TΓ2−1W^˙2=V˙1+z2g2x−2f2x−2+g2x−2x3+d2−α˙1+g˙2x−2z222g22x−2+W~2TΓ2−1W^˙2=V˙1+z2z3+α2+f2x−2−α˙1g2x−2+z2d2g2x−2+g˙2x−2z222g22x−2+W~2TΓ2−1W^˙2.

According to Assumption 1 we can get

(21) V˙2V˙1+z2z3+α2+f2x−2−α˙1g2x−2+z22ρ222g22x−2+P2∗22+g˙2x−2z222g22x−2+W~2TΓ2−1W^˙2=V˙1+z2z3+α2+f2x−2−α˙1g2x−2+z2ρ222g22x−2+P2∗22+g˙2x−2z222g22x−2+W~2TΓ2−1W^˙2.

There is an ideal feedback control law:

(22) $$\begin{array}{c} \alpha_{2}^{*}=-z_{1}-c_{2}z_{2}-\left[\;\frac{f_{2}\left({\overset{-}{x}}_{2}\right)-{\dot{\alpha }}_{1}}{g_{2}\left({\overset{-}{x}}_{2}\right)}+\frac{z_{2}^{}\rho_{2}^{2}}{2g_{2}^{2}\left({\overset{-}{x}}_{2}\right)}\;\right], \end{array}$$

where *c*_2_ > 0 is a designed controller parameter.

Because of the unknown smooth functions $f_{2}({\overset{-}{x}}_{2})$ and $g_{2}({\overset{-}{x}}_{2})$, we cannot actually get the ideal feedback control law *α*_2_^*∗*^; from (22) we can see that the unknown part is a smooth function of ${\overset{-}{x}}_{2}$ and ${\dot{\alpha }}_{1}$; let

(23) $$\begin{array}{c} h_{2}\left(\;Z_{2}\;\right)≜\frac{f_{2}\left({\overset{-}{x}}_{2}\right)-{\dot{\alpha }}_{1}}{g_{2}\left({\overset{-}{x}}_{2}\right)}+\frac{z_{2}^{}\rho_{2}^{2}}{2g_{2}^{2}\left({\overset{-}{x}}_{2}\right)}, \end{array}$$

where $Z_{2}≜{\left[{\overset{-}{x}}_{2}^{T},(\partial \alpha_{1}/\partial x_{1}),\phi_{1}\right]}^{T}\subset R^{4}$. RBF neural network *W*_2_^*T*^ *S*_2_(*Z*_2_) is used to approximate the unknown function *h*_2_(*Z*_2_), and *α*_2_^*∗*^ can be expressed as

(24) $$\begin{array}{c} \alpha_{2}^{*}=-z_{1}-c_{2}z_{2}-W_{2}^{*T}S_{2}\left(\;Z_{2}\;\right)-e_{2}, \end{array}$$

where *W*_2_^*∗*^ is expressed as the ideal constant weight vector and |*e*_2_| ≤ *e*_2_^*∗*^ is the estimated error and meets *e*_2_^*∗*^ > 0.

Because *W*_2_^*∗*^ is unknown, select the following virtual control law:

(25) $$\begin{array}{c} \alpha_{2}=-z_{1}-c_{2}z_{2}-{\hat{W}}_{2}^{T}S_{2}\left(\;Z_{2}\;\right), \end{array}$$

where ${\hat{W}}_{2}$ is the estimated value of *W*_2_^*∗*^; then

(26) V˙2≤V˙1−z1z2+z2z3−c2z22+g˙2x−2z222g22x−2+z2e2+P2∗22−W~2TS2z2+W~2TΓ2−1W^˙2,

where ${\tilde{W}}_{2}={\hat{W}}_{2}-W_{2}^{*}$.

Adaptive law can be chosen as

(27) $$\begin{array}{c} {\dot{\hat{W}}}_{2}={\dot{\tilde{W}}}_{2}=\Gamma_{2}\left[\;S_{2}\left(\;Z_{2}\;\right)z_{2}-\sigma_{2}{\hat{W}}_{2}\;\right], \end{array}$$

where *σ*_2_ > 0; then

(28) V˙2≤V˙1−z1z2+z2z3−c2z22+g˙2x−2z222g22x−2+z2e2+P2∗22−σ2W~2TW^2.

Let *c*_2_ = *c*_20_ + *c*_21_,  *c*_20_, *c*_21_ > 0; then the upper equation becomes

(29) V˙2≤V˙1−z1z2+z2z3−c20+g˙2x−22g22x−2z22−c21z22+z2e2+P2∗22−σ2W~2TW^2.

According to the complete square formula,

(30) −σ2W~2TW^2−σ2W~2TW~2+W2∗≤−σ2W~22+σ2W~2W2∗≤−σ2W~222+σ2W2∗22,−c21z22+z2e2−c21z22+z2e2≤e224c21≤e2∗24c21.

Because $-(c_{20}+({\dot{g}}_{2}/2g_{2}^{2}))z_{2}^{2}\leq -(c_{20}-(g_{2d}/2g_{2m}^{2}))z_{2}^{2}$, then we can make (*c*_20_^*∗*^≜*c*_20_ − (*g*_2*d*_/2*g*_2*m*_^2^)) > 0 by selecting the proper *c*_20_; then

(31) V˙2V˙1−z1z2+z2z3−c20∗z22−σ2W~222+σ2W2∗22+e2∗24c21+P2∗22≤z2z3−∑k=12ck0∗zk2−∑k=12σkW~k22+∑k=12σkWk∗22+∑k=12ek∗24ck1.

The cross coupling *z*_2_ *z*_3_ in (31) will be eliminated in the next step.

*Step i*  (3 ≤ *i* ≤ *n* − 1). The derivative of *z*_*i*_ = *x*_*i*_ − *α*_*i*−1_ can be calculated as

(32) $$\begin{array}{c} {\dot{z}}_{i}=f_{i}\left(\;{\overset{-}{x}}_{i}\;\right)+g_{i}\left(\;{\overset{-}{x}}_{i}\;\right)x_{i+1}-{\dot{\alpha }}_{i-1}, \end{array}$$

where

(33) α˙i−1=∑k=1i−1∂αi−1∂xkgkx−kxk+1+fkx−k+φi−1,ϕi−1=∑k=1i−1∂αi−1∂xdx˙d+∑k=1i−1∂αi−1∂W^kΓkSkZkzk−σkW^k.

Consider the following Lyapunov function:

(34) $$\begin{array}{c} V_{i}=V_{i-1}+\frac{1}{2g_{i}\left({\overset{-}{x}}_{i}\right)}z_{i}^{2}+\frac{1}{2}{\tilde{W}}_{i}^{T}\Gamma_{i}^{-1}{\tilde{W}}_{i}, \end{array}$$

where Γ_*i*_ = Γ_*i*_^*T*^ > 0 is an adaptive gain matrix.

Then the derivation of *V*_*i*_ can be calculated as

(35) V˙iV˙i−1+ziz˙igix−i+g˙ix−izi22gi2x−i+W~iTΓi−1W^˙i=V˙i−1+zigix−ifix−i+gix−ixi+1+di−α˙i−1+g˙ix−izi22gi2x−i+W~iTΓi−1W^˙i=V˙i−1+zizi+1+αi+fix−i−α˙i−1gix−i+zidigix−i+g˙ix−izi22gi2x−i+W~iTΓi−1W^˙i.

According to Assumption 1 we can get

(36) V˙iV˙i−1+zizi+1+αi+fix−i−α˙i−1gix−i+zi2ρi22gi2x−i+Pi∗22+g˙ix−izi22gi2x−i+W~iTΓi−1W^˙i=V˙i−1+zizi+1+αi+fix−i−α˙i−1gix−i+zi2ρi22gi2x−i+Pi∗22+g˙ix−izi22gi2x−i+W~iTΓi−1W^˙i.

There is an ideal feedback control law as

(37) $$\begin{array}{c} \alpha_{i}^{*}=-z_{i-1}-c_{i}z_{i}-\left[\;\frac{f_{i}\left({\overset{-}{x}}_{i}\right)-{\dot{\alpha }}_{i-1}}{g_{i}\left({\overset{-}{x}}_{i}\right)}+\frac{z_{i}^{}\rho_{i}^{2}}{2g_{i}^{2}\left({\overset{-}{x}}_{i}\right)}\;\right], \end{array}$$

where *c*_*i*_ > 0 is designed controller parameter.

Because of the unknown smooth functions $f_{i}({\overset{-}{x}}_{i})$ and $g_{i}({\overset{-}{x}}_{i})$, we cannot actually get the ideal feedback control law *α*_*i*_^*∗*^; from (37) we can see that the unknown part is a smooth function of ${\overset{-}{x}}_{i}$ and ${\dot{\alpha }}_{i-1}$, and let

(38) $$\begin{array}{c} h_{i}\left(\;Z_{i}\;\right)≜\frac{f_{i}\left({\overset{-}{x}}_{i}\right)-{\dot{\alpha }}_{i-1}}{g_{i}\left({\overset{-}{x}}_{i}\right)}+\frac{z_{i}^{}\rho_{i}^{2}}{2g_{i}^{2}\left({\overset{-}{x}}_{i}\right)}, \end{array}$$

where

(39) $$\begin{array}{c} Z_{i}≜{\left[\;{\overset{-}{x}}_{i}^{T},\frac{\partial \alpha_{i-1}}{\partial x_{1}},\ldots ,\frac{\partial \alpha_{i-1}}{\partial x_{i-1}},\varphi_{i-1}\;\right]}^{T}\subset R^{2i}. \end{array}$$

By introducing the direct variable (∂*α*_*i*−1_/∂*x*_1_),…, (∂*α*_*i*−1_/∂*x*_*i*−1_), *φ*_*i*−1_, we can make the number of neural networks minimized. RBF neural network *W*_*i*_^*T*^ *S*_*i*_(*Z*_*i*_) is used to approximate the unknown function *h*_*i*_(*Z*_*i*_), and *α*_*i*_^*∗*^ can be expressed as

(40) $$\begin{array}{c} \alpha_{i}^{*}=-z_{i-1}-c_{i}z_{i}-W_{i}^{*T}S_{i}\left(\;Z_{i}\;\right)-e_{i}, \end{array}$$

where |*e*_*i*_| ≤ *e*_*i*_^*∗*^ is estimated error and meets *e*_*i*_^*∗*^ > 0.

Because *W*_*i*_^*∗*^ is unknown, select the following virtual control law:

(41) $$\begin{array}{c} \alpha_{i}=-z_{i-1}-c_{i}z_{i}-{\hat{W}}_{i}^{T}S_{i}\left(\;Z_{i}\;\right), \end{array}$$

where *W*_*i*_^*∗*^ is the estimated value of ${\hat{W}}_{i}$; then

(42) V˙i≤V˙i−1−zi−1zi+zizi+1−cizi2+g˙ix−izi22gi2x−i+ziei+Pi∗22−W~iTSizi+W~iTΓi−1W^˙i,

where ${\tilde{W}}_{i}={\hat{W}}_{i}-W_{i}^{*}$.

The following adaptive law can be selected as

(43) $$\begin{array}{c} {\dot{\hat{W}}}_{i}={\dot{\tilde{W}}}_{i}=\Gamma_{i}\left[\;S_{i}\left(\;Z_{i}\;\right)z_{i}-\sigma_{i}{\hat{W}}_{i}\;\right], \end{array}$$

where *σ*_*i*_ > 0; then

(44) V˙i≤V˙i−1−zi−1zi+zizi+1−cizi2+g˙ix−izi22gi2x−i+ziei+Pi∗22−σiW~iTW^i.

Let *c*_*i*_ = *c*_*i*0_ + *c*_*i*1_,  *c*_*i*0_, *c*_*i*1_ > 0; then (44) can be rewritten as

(45) V˙i≤V˙i−1−zi−1zi+zizi+1−ci0+g˙ix−i2gi2x−izi2−ci1zi2+ziei+Pi∗22−σiW~iTW^i.

According to the complete square formula,

(46) −σiW~iTW^i−σiW~iTW~i+Wi∗≤−σiW~i2+σiW~iWi∗≤−σiW~i22+σiWi∗22,−ci1zi2+ziei−ci1zi2+ziei≤ei24ci1≤ei∗24ci1.

Because $-(c_{i0}+({\dot{g}}_{i}/2g_{i}^{2}))z_{i}^{2}\leq -(c_{i0}-(g_{id}/2g_{im}^{2}))z_{i}^{2}$, then we can make (*c*_*i*0_^*∗*^≜*c*_*i*0_ − (*g*_*id*_/2*g*_*im*_^2^)) > 0 by selecting the proper *c*_*i*0_; then

(47) V˙iV˙i−1−zi−1zi+zizi+1−ci0∗zi2−σiW~i22+σiWi∗22+ei∗24ci1+Pi∗22≤zizi+1−∑k=1ick0∗zk2−∑k=1iσkW~k22+∑k=1iσkWk∗22+∑k=1iek∗24ck1+∑k=1iPk∗22.

The cross coupling *z*_*i*_ *z*_*i*+1_ in (47) will be eliminated in the next step.

*Step n*. The derivative of *z*_*n*_ = *x*_*n*_ − *α*_*n*−1_ can be calculated as

(48) $$\begin{array}{c} {\dot{z}}_{n}=f_{n}\left(\;{\overset{-}{x}}_{n}\;\right)+g_{n}\left(\;{\overset{-}{x}}_{n-1}\;\right)u-{\dot{\alpha }}_{n-1}, \end{array}$$

where

(49) $$\begin{array}{c} {\dot{\alpha }}_{n-1}=\overset{n-1}{\underset{k=1}{\sum }}\frac{\partial \alpha_{n-1}}{\partial x_{k}}\left(\;g_{k}\left(\;{\overset{-}{x}}_{k}\;\right)x_{k+1}+f_{k}\left(\;{\overset{-}{x}}_{k}\;\right)\;\right)+\phi_{n-1}, \end{array}$$

where

(50) ϕn−1=∑k=1n−1∂αn−1∂xdx˙d+∑k=1n−1∂αn−1∂W^kΓkSkZkzk−σkW^k.

Consider the following Lyapunov function:

(51) $$\begin{array}{c} V_{n}=V_{n-1}+\frac{1}{2g_{n}\left({\overset{-}{x}}_{n}\right)}z_{n}^{2}+\frac{1}{2}{\tilde{W}}_{n}^{T}\Gamma_{n}^{-1}{\tilde{W}}_{n}, \end{array}$$

where Γ_*n*_ = Γ_*n*_^*T*^ > 0 is an adaptive gain matrix. Then the derivation of *V*_*n*_ can be calculated as

(52) V˙nV˙n−1+znz˙ngix−i+g˙nx−nzn22gn2x−n+W~nTΓn−1W^˙n=V˙n−1+zngnx−nfnx−n+gnx−nu+dn−α˙n−1+g˙nx−nzn22gn2x−n+W~nTΓn−1W^˙n=V˙n−1+znzn+1+u+fnx−n−α˙n−1gnx−n+zndngnx−n+g˙nx−nzn22gn2x−n+W~nTΓn−1W^˙n.

According to Assumption 1 we can get

(53) V˙nV˙n−1+znzn+1+u+fix−i−α˙n−1gix−i+zn2ρn22gn2x−n+Pn∗22+g˙nx−nzn22gn2x−n+W~nTΓn−1W^˙n=V˙n−1+znzn+1+u+fnx−n−α˙n−1gnx−n+zn2ρn22gn2x−n+Pn∗22+g˙nx−nzn22gn2x−n+W~nTΓn−1W^˙n.

There is an ideal feedback control law as

(54) $$\begin{array}{c} u^{*}=-z_{i-1}-c_{i}z_{i}-\left[\;\frac{f_{i}\left({\overset{-}{x}}_{i}\right)-{\dot{\alpha }}_{i-1}}{g_{i}\left({\overset{-}{x}}_{i}\right)}+\frac{z_{i}^{}\rho_{i}^{2}}{2g_{i}^{2}\left({\overset{-}{x}}_{i}\right)}\;\right], \end{array}$$

where *c*_*n*_ > 0 is designed controller parameter.

Because of the unknown smooth functions $f_{n}({\overset{-}{x}}_{n})$ and $g_{i}({\overset{-}{x}}_{i})$, we cannot actually get the ideal feedback control law *u* ^*∗*^; from (54) we can see the unknown part is a smooth function of ${\overset{-}{x}}_{n}$ and ${\dot{\alpha }}_{n-1}$, and let

(55) $$\begin{array}{c} h_{n}\left(\;Z_{i}\;\right)≜\frac{f_{n}\left({\overset{-}{x}}_{n}\right)-{\dot{\alpha }}_{n-1}}{g_{n}\left({\overset{-}{x}}_{n}\right)}+\frac{z_{n}\rho_{n}^{2}}{2g_{n}^{2}\left({\overset{-}{x}}_{n}\right)}, \end{array}$$

where $Z_{n}≜{\left[{\overset{-}{x}}_{n}^{T},\partial \alpha_{n-1}/\partial x_{1},\ldots ,\partial \alpha_{n-1}/\partial x_{n-1},\phi_{n-1}\right]}^{T}\subset R^{2n}$.

RBF neural network *W*_*n*_^*T*^ *S*_*n*_(*Z*_*n*_) is used to approximate the unknown function *h*_*n*_(*Z*_*n*_), and *u* ^*∗*^ can be expressed as

(56) $$\begin{array}{c} u^{*}=-z_{n-1}-c_{n}z_{n}-W_{n}^{*T}S_{n}\left(\;Z_{n}\;\right)-e_{n}, \end{array}$$

where |*e*_*n*_| ≤ *e*_*n*_^*∗*^ is estimated error and meets *e*_*n*_^*∗*^ > 0.

Because *W*_*n*_^*∗*^ is unknown, select the following virtual control law:

(57) $$\begin{array}{c} u=-z_{n-1}-c_{n}z_{n}-{\hat{W}}_{n}^{T}S_{n}\left(\;Z_{n}\;\right), \end{array}$$

where ${\hat{W}}_{i}$ is the estimated value of *W*_*i*_^*∗*^; then

(58) V˙n≤V˙n−1−zn−1zn+znzn+1−cnzn2+g˙nx−nzn22gn2x−n+znen+Pn∗22−W~nTSnzn+W~nTΓn−1W^˙n,

where ${\tilde{W}}_{n}={\hat{W}}_{n}-W_{n}^{*}$.

The following adaptive law can be selected as

(59) $$\begin{array}{c} {\dot{\hat{W}}}_{n}={\dot{\tilde{W}}}_{n}=\Gamma_{n}\left[\;S_{n}\left(\;Z_{n}\;\right)z_{n}-\sigma_{n}{\hat{W}}_{n}\;\right], \end{array}$$

where *σ*_*n*_ > 0; then

(60) V˙n≤V˙n−1−zn−1zn+znzn+1−cnzn2+g˙nx−nzn22gn2x−n+znen+Pn∗22−σnW~nTW^n.

Let *c*_*n*_ = *c*_*n*0_ + *c*_*n*1_,  *c*_*n*0_, *c*_*n*1_ > 0; (60) can be rewritten as

(61) V˙n≤V˙n−1−zn−1zn+znzn+1−cn0+g˙nx−n2gn2x−nzn2−cn1zn2+znen+Pn∗22−σnW~nTW^n.

According to the complete square formula,

(62) −σnW~nTW^n−σnW~nTW~n+Wn∗≤−σnW~n2+σnW~nWn∗≤−σnW~n22+σnWn∗22,−cn1zn2+znen−cn1zn2+znen≤en24cn1≤en∗24cn1.

Because $-(c_{n0}+({\dot{g}}_{n}/2g_{n}^{2}))z_{n}^{2}\leq -(c_{n0}-(g_{nd}/2g_{nm}^{2}))z_{n}^{2}$, then we can make (*c*_*n*0_^*∗*^≜*c*_*n*0_ − (*g*_*nd*_/2*g*_*nm*_^2^)) > 0 by selecting the proper *c*_*n*0_; then

(63) V˙n≤−∑k=1nck0∗zk2−∑k=1nσkW~k22+∑k=1nσkWk∗22+∑k=1nek∗24ck1+∑k=1nPk∗22.

Let *δ*≜∑_*k*=1_^*n*^(*σ*_*k*_‖*W*_*k*_^*∗*^‖^2^/2) + ∑_*k*=1_^*n*^(*e*_*k*_^*∗*2^/4*c*_*k*1_) + ∑_*k*=1_^*n*^(*p*_*k*_^*∗*2^/2),  *c*_*k*0_^*∗*^ ≥ (*γ*/2*g*_*km*_),  *c*_*k*0_ > (*γ*/2*g*_*km*_)+(*g*_*kd*_/2*g*_*km*_^2^),  *k* = 1,2,…, *n*,  where *γ* > 0,  *σ*_*k*_ ≥ *γλ*_max_{Γ_*k*_^−1^},  *k* = 1,2,…, *n*; then

(64) V˙n−∑k=1nck0∗zk2−∑k=1nσkW~k22+δ≤−∑k=1nγ2gkmzk2−∑k=1nγW~kTΓk−1W~k2gkm+δ≤−γ∑k=1n12gkzk2+∑k=1nW~kTΓk−1W~k2+δ≤−γVn+δ.

The stability and control performance of the closed-loop adaptive system are demonstrated by the following theorem.

**Theorem 2 .**

In the initial conditions, by formula (1), reference model (2), control law (57), and neural network weight update rate in (12), (27), (43), and (59), supposing that there is a large enough set of closed sets *Ω*_*i*_ ∈ *R* ^2*i*^,  *i* = 1,2,…, *n*, for any given moment *t* ≥ 0, making *Z*_*i*_ ∈ *Ω*_*i*_, the following conclusions can be obtained as follows:

(1) The signal of the whole closed-loop system is bounded, and the state variable ${\overset{-}{x}}_{n}$ and the neural network estimation errors ${\hat{W}}_{1}^{T},\ldots ,{\hat{W}}_{n}^{T}$ will eventually converge to the closed set as follows:
  (65) $$\begin{array}{c} \Omega_{s1}≜\left\{\;{\overset{-}{x}}_{n},{\hat{W}}_{1},\ldots ,{\hat{W}}_{n}\;|\;V<\frac{\delta }{\gamma },\;x_{d}\in \Omega_{d}\;\right\}. \end{array}$$
(2) By choosing the proper control parameters, the output tracking error *y*(*t*) − *y*_*d*1_(*t*) is close to a small neighborhood of zero [21].

## 3. Adaptive Robust Neural Network Control for Ship Course

### 3.1. Problem Formulation

This section introduces a simplified dynamic model of an underactuated surface vehicle with only one control input *δ* for heading control. A surface ship usually has three degrees of freedom for path following control in horizontal plane. Assuming that the vessel has three planes of symmetry, for most underactuated vessels have port/starboard symmetry, it can be neglected to simplify the vessel model for controller design. The detailed model which considers the environment disturbances can be set as follows:

(66) y˙=Usin⁡ψ,ψ˙=r,r˙=−1Tr−αTr3+KTδ+Δ,y1=y,y2=ψ,

where *y* denotes transverse displacement in the earth inertial coordinates; $U=\sqrt{u^{2}+v^{2}}$ is resultant velocity of ship; *ψ* is course angle; *r* is yawing angular velocity; *K*, *T* represent performance index for ship steering; *α* is coefficient of nonlinear term; *δ* is control rudder angle; *y*_1_, *y*_2_ represent system output.

The control objective is to design the controller *δ* to make the control output *y*, *ψ* achieve the setting value (*y*_*d*_, *ψ*_*d*_). Because the dimension of the system control input is less than the degree of freedom of the system, it is an underactuated system.

### 3.2. Dynamic Controller Design

Selection of coordinate transformation is as follows:

(67) $$\begin{array}{c} w_{e}=\psi +\arcsin\left(\;\frac{ky}{\sqrt{1+{\left(ky\right)}^{2}}}\;\right). \end{array}$$

The original system can be transformed into a single input single output system:

(68) x˙1=ky˙1+ky2+x2,x˙2=−a1x2−a2x23+bu+Δ,

where *a*_1_ = 1/*T*,  *a*_2_ = *α*/*T*,  *b* = *K*/*T*,  *x*_1_ = *w*_*e*_,  *x*_2_ = *r*,  *u* = *δ*, and the output of whole system is *x*_1_.

For system model (67) and (68), the controller design is carried out by using backstepping method.

**Step 1 .**

Let *z*_1_ = *x*_1_,  *x*_*d*1_ = 0; then

(69) $$\begin{array}{c} {\dot{z}}_{1}=\frac{k\dot{y}}{1+{\left(ky\right)}^{2}}+x_{2}. \end{array}$$

For the subsystem *z*_1_,  *α*_1_^*∗*^≜*x*_2_ is chosen as virtual control input. Select the Lyapunov function *V*_*z*1_ = (1/2)*z*_1_^2^, and there is

(70) $$\begin{array}{c} {\dot{V}}_{z1}=z_{1}{\dot{z}}_{1}=\left(\;\frac{k\dot{y}}{1+{\left(ky\right)}^{2}}+x_{2}\;\right)z_{1}. \end{array}$$

Let *z*_2_ = *x*_2_ − *α*_1_; then *x*_2_ = *z*_2_ + *α*_1_,

(71) $$\begin{array}{c} {\dot{V}}_{z1}=\left(\;\frac{k\dot{y}}{1+{\left(ky\right)}^{2}}+z_{2}+\alpha_{1}\;\right)z_{1}. \end{array}$$

Select the following virtual control law:

(72) $$\begin{array}{c} \alpha_{1}^{*}=-c_{1}z_{1}-\frac{k\dot{y}}{1+{\left(ky\right)}^{2}}. \end{array}$$

${\dot{V}}_{z1}=z_{1}z_{2}-c_{1}z_{1}^{2}$, because $k\dot{y}/\left(1+(ky{)}^{2}\right)$ is unknown function, $h_{1}\left(Z_{1}\right)=k\dot{y}/\left(1+(ky{)}^{2}\right)$, and we will adopt RBF NN to estimate *h*_1_(*Z*_1_) and get *h*_1_(*Z*_1_) = *W*_1_^*∗T*^ *S*_1_(*Z*_1_) + *ε*_1_. But the actual use of the NN for the system is $h_{1}\left(Z_{1}\right)={\hat{W}}_{1}^{T}S_{1}\left(Z_{1}\right)$. Actual virtual control input is $\alpha_{1}=-c_{1}z_{1}-{\hat{W}}_{1}^{T}S_{1}\left(Z_{1}\right)$; then

(73) z˙1ky˙1+ky2+z2+α1=z2−c1z1−W~1S1Z1+ε1,

where ${\tilde{W}}_{1}={\hat{W}}_{1}-W_{1}^{*}$.

Select Lyapunov function as

(74) $$\begin{array}{c} V_{1}=V_{z1}+\frac{1}{2}{\tilde{W}}_{1}^{T}\Gamma^{-1}{\tilde{W}}_{1}; \end{array}$$

then

(75) V˙1V˙z1+W~1Γ−1W^˙1≤z1z2+α1+h1Z1=z1z2−c1z1−W^1S1Z1+W1∗S1Z1+ε1+W~1Γ−1W^˙1=z1z2−c1z1−W~1S1Z1+ε1+W~1Γ−1W^˙1.

The adaptive law of neural network can be designed as

(76) $$\begin{array}{c} {\dot{\hat{W}}}_{1}={\tilde{W}}_{1}=\Gamma_{1}\left[\;S_{1}\left(\;Z_{1}\;\right)z_{1}-\sigma_{1}{\hat{W}}_{1}\;\right], \end{array}$$

where *σ*_1_ > 0. Let *c*_1_ = *c*_10_ + *c*_11_, where *c*_10_, *c*_11_ > 0.

Furthermore,

(77) $$\begin{array}{c} {\dot{V}}_{1}=z_{1}z_{2}-c_{10}z_{1}^{2}-c_{11}z_{1}^{2}+z_{1}\varepsilon_{1}-\sigma_{1}{\tilde{W}}_{1}^{T}{\hat{W}}_{1}; \end{array}$$

then

(78) −σ1W~1TW^1−σ1W~1TW~1+W1∗≤−σ1W~12+σ1W~1W1∗≤−σ1W~122+σ1W1∗22

because

(79) $$\begin{array}{c} -c_{11}z_{1}^{2}+z_{1}\varepsilon_{1}\leq -c_{11}z_{1}^{2}+z_{1}\left|\;\varepsilon_{1}\;\right|\leq \frac{\varepsilon_{1}^{2}}{4c_{11}}\leq \frac{\varepsilon_{1}^{*2}}{4c_{11}}. \end{array}$$

Finally we can get

(80) $$\begin{array}{c} {\dot{V}}_{1}<z_{1}z_{2}-c_{10}^{*}z_{1}^{2}-\frac{\sigma_{1}{\left\|{\tilde{W}}_{1}\right\|}^{2}}{2}+\frac{\sigma_{1}{\left\|W_{1}^{*}\right\|}^{2}}{2}+\frac{\varepsilon_{1}^{*2}}{4c_{11}}. \end{array}$$

**Step 2 .**

Let *z*_2_ = *x*_2_ − *α*_1_; derivation of *z*_2_ can be calculated as

(81) z˙2f2x−2+g2x−2u+Δ−α˙1=−a1x2−a2x23+bu+Δ−α˙1.

Because *V*_*z*2_ = (1/2*b*)*z*_2_^2^, then

(82) V˙z21bz2z˙2=1bz2−a1x2−a2x23+bu+Δ−α˙1=z2u+1b−a1x2−a2x23−α˙1+Δbz2≤z2u+1b−a1x2−a2x23−α˙1+ρ2z22b+p22,

where Δ ≤ *p* · *ρ*(*x*), *p* is unknown parameter, *ρ*(*x*) is known nonlinear function, and then

(83) $$\begin{array}{c} u^{*}=-z_{1}-c_{2}z_{2}-\frac{1}{b}\left(\;-a_{1}x_{2}-a_{2}{x_{2}}^{3}-{\dot{\alpha }}_{1}+\frac{\rho^{2}z_{2}}{2b}\;\right). \end{array}$$

Let

(84) $$\begin{array}{c} h_{2}\left(\;Z_{2}\;\right)=\frac{1}{b}\left(\;-a_{1}x_{2}-a_{2}{x_{2}}^{3}-{\dot{\alpha }}_{1}+\frac{\rho^{2}z_{2}}{2b}\;\right). \end{array}$$

Equation (83) can be rewritten as

(85) $$\begin{array}{c} u^{*}=-z_{1}-c_{2}z_{2}-h_{2}\left(\;Z_{2}\;\right). \end{array}$$

In the same way we use RBF NN estimate *h*_2_(*Z*_2_):

(86) $$\begin{array}{c} h_{2}\left(\;Z_{2}\;\right)={W_{2}^{*}}^{T}S_{2}\left(\;Z_{2}\;\right)+\varepsilon_{2}. \end{array}$$

The actual use of the NN for the system and controller can be expressed as

(87) h2Z2=W^2TS2Z2,u=z1−c2z2−W^2TS2Z2.

Select Lyapunov function as

(88) $$\begin{array}{c} V_{2}=V_{1}+V_{z2}+\frac{1}{2}{\tilde{W}}_{2}^{T}\Gamma^{-1}{\tilde{W}}_{2}. \end{array}$$

The derivation of *V*_2_ can be calculated as

(89) V˙2=V˙1+V˙z2+W~1Γ−1W^˙1≤z1z2−c10∗z12−σ1W~122+σ1W1∗22+ε1∗24c11+z2−z1−c2z2−W^2S2Z2+W2∗S2Z2+ε2+p22+W~1Γ−1W^˙1=−∑i=12ci0∗zi2−∑i=12σiW~i22+∑i=12σiWi∗22+∑i=12εi∗24c11+p22.

Therefore, all signals in the close loop of course tracking system are stable, and the tracking errors can be made arbitrarily small by selecting appropriate controller parameters. So the final control law can be designed as

(90) $$\begin{array}{c} u=z_{1}-c_{2}z_{2}-{\hat{W}}_{2}^{T}S_{2}\left(\;Z_{2}\;\right). \end{array}$$

## 4. Numerical Simulations and Analysis

The simulation experiment can be operated based on an experimental ship. The nonlinear mathematical model for the ship has been presented in [22], which captures the fundamental characteristics of dynamics and offers good maneuverability in the open-loop test. To illustrate the effectiveness of the theoretical results, the proposed control scheme is implemented and simulated with the above nonlinear model with tracking task.

The characteristic parameters of the ship used in the simulation are given as *K* = 0.478, *T* = 216, and *α* = 30. Neural network contains 25 neurons; that is, *l*_1_ = 25; the center vector *μ*_*l*_  (*l* = 1,2,…, *l*_1_) is uniformly distributed in the width [−2,2] × [−2,2] × [−2,2]. Neural network ${\hat{W}}_{2}^{T}S_{2}(Z_{2})$ contains 135 neurons; that is, *l*_2_ = 125; the center vector *μ*_*l*_  (*l* = 1,2,…, *l*_2_) is uniformly distributed in the width [−4,4] × [−4,4] × [−4,4] × [−4,4] × [−4,4] × [−4,4] × [−4,0] × [−6,6]. The controller design parameters are given as follows which satisfy the condition mentioned in design procedure: *k* = 0.1394, *c*_1_ = 4, *c*_2_ = 120, Γ_1_ = diag⁡{3}, Γ_2_ = diag⁡{4}, and *σ*_1_ = 4, *σ*_2_ = 2. The initial linear and angular velocity of ship used in the simulation are given as [*u*, *v*, *r*]^*T*^ = [0.1,0, 0]^*T*^,  [*x*, *y*, *ψ*]^*T*^ = [10,30, −*π*/4]^*T*^ is the initial position and orientation vector of ship, and the desired velocity of ship is given as *u*_*d*_ = 1 (m/s). We choose the reference trajectory as 10cos⁡*ωt*.

**Figure 1:**
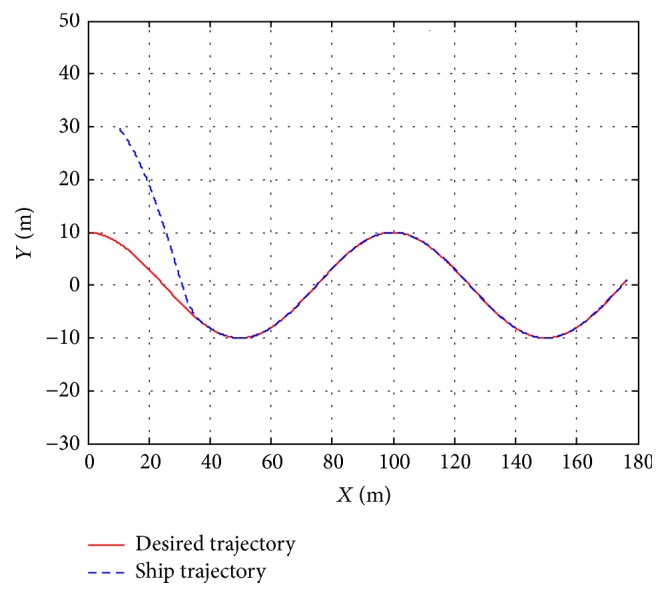
Ship tracking performance of proposed control method.

**Figure 2 fig2:**
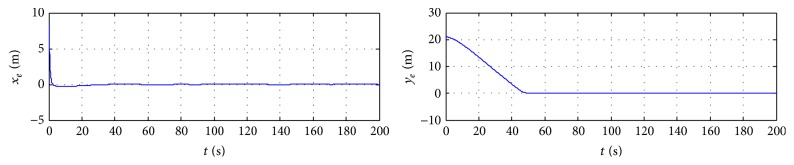
Tracking errors of surge and sway.

**Figure 3 fig3:**
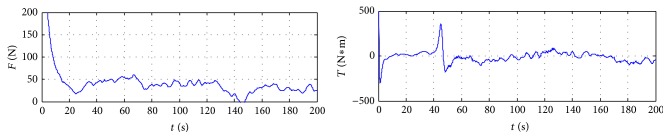
Control force and torque of ship.

**Figure 4:**
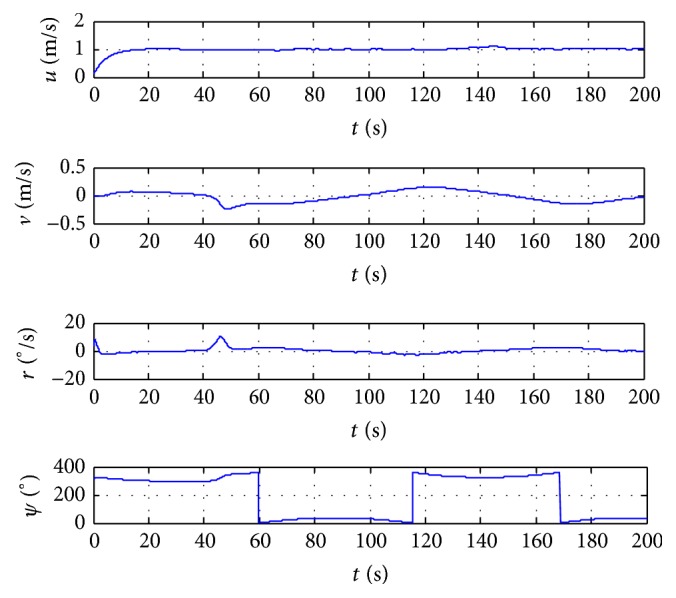
State changing curves of ship.

In order to further verify the validity of the proposed control method, the algorithm of this paper is compared with the simulation results in [12]. So the robustness of trajectory tracking controller against the disturbance and model uncertainties can be evaluated. All the simulation results are depicted in Figures 1 [Fig fig2] [Fig fig3]–4.  Figure 1 shows the trajectory tracking of ship with the given path, and the ship can track and converge to the reference path with more accuracy in [12].  Figure 2 plots the position tracking errors; the along-track and cross-track errors asymptotically converge to zero faster.  Figure 3 gives the control inputs response. Surge, sway, yaw velocities, and orientation of ship during the trajectory tracking control process are plotted in Figure 4, which gives a clear insight into the model response involved in nonlinear dynamics.

## 5. Conclusions

In this paper, we proposed a solution to the course control of underactuated surface vessel. Firstly, the direct adaptive neural network control and its application are introduced. Then the backstepping controller with robust neural network is designed to deal with the uncertain and underactuated characteristics for the ship. Neural networks are adopted to determine the parameters of the unknown part of the ideal virtual control and the ideal control; even the weights of neural network are updated by using adaptive technique. Finally uniform stability for the convergence of tracking errors has been proven through Lyapunov stability theory. The simulation results illustrate the performance of the proposed course tracking controller with good precision.
